# ﻿Four new species of the genus *Yunguirius* (Araneae, Agelenidae) from China

**DOI:** 10.3897/zookeys.1211.126487

**Published:** 2024-09-02

**Authors:** Mian Wei, Jie Liu, Kai Wang

**Affiliations:** 1 Hubeiate Key Laboratory of Regional Development and Environmental Response, Faculty of Re-sources and Environmental Science, Hubei University, Wuhan 430062, China; 2 The State Key Laboratory of Biocatalysis and Enzyme Engineering of China, School of Life Sciences, Hubei University, Wuhan 430062, Hubei, China; 3 School of Nuclear Technology and Chemistry and Biology, Hubei University of Science and Technology, Xianning 437100, Hubei, China

**Keywords:** Biodiversity, coelotine spiders, description, morphology, taxonomy

## Abstract

Four new species of *Yunguirius* B. Li, Zhao & S.Q. Li, 2023 are described from China, namely: *Yunguiriusparvus* Wei & Liu, **sp. nov.** (♀), *Yunguiriustrigonus* Wei & Liu, **sp. nov.** (♀), *Yunguiriuswangqiqiae* Wei & Liu, **sp. nov.** (♀), and *Yunguiriusxiannushanensis* Wei & Liu, **sp. nov.** (♀).

## ﻿Introduction

Coelotinae F.O. Pickard-Cambridge, 1893, the most diverse subfamily of Agelenidae C.L. Koch, 1837, is endemic to the Northern Hemisphere. To date, 806 species across 40 genera have been described (WSC 2024). In recent years, there has been frequent reporting of new taxa as well as taxonomic revisions of previously described species, particularly those in the genera *Coelotes* Blackwall, 1841 and *Draconarius* Ovtchinnikov, 1999 (Chen et al. 2016; Chen 2017; Li et al. 2018a, 2018b, 2019, 2023; Okumura 2020; Okumura et al. 2021; Okumura and Zhao 2022; Hoang et al. 2023; Luo et al. 2023). The genus *Yunguirius*, was recently described by Li et al. (2023) based on *Draconariusornatus* (Wang, Yin, Peng & Xie, 1990) and includes two newly described species along with two others transferred from *Draconarius*: *Y.duoge* B. Li, Zhao & S.Q. Li, 2023, *Y.subterebratus* (Zhang, Zhu & Wang, 2017), *Y.terebratus* (Peng & Wang, 1997) and *Y.xiangding* B. Li, Zhao & S.Q. Li, 2023. According to previous studies, all five described *Yunguirius* species predominantly occur along the northern edge of the Yunnan-Guizhou Plateau.

While examining our specimens, four undescribed species of *Yunguirius* collected from the northern edge of the Yunnan-Guizhou Plateau were discovered. We report these new species in the current paper, the descriptions, detailed colour illustrations, and distributional maps of new species are provided.

### ﻿Materials and methods

All specimens were preserved in 75% ethanol and examined with an Olympus SZX7 stereomicroscope. Male palps and female genitalia were dissected from the spider bodies to be examined and photographed. Epigynes were cleared with Proteinase K to study their inner structures. Photographs were taken with a Canon EOS 90D wide zoom digital camera (8.5 megapixels) mounted on an Olympus BX 43 compound microscope. The images were montaged using Helicon Focus 7.0.2 image stacking software. Left palps are illustrated. Leg measurements are given as total length (coxa, trochanter, femur, patella, tibia, metatarsus, tarsus). Only the structures on the left (e.g., pedipalpus, legs) were measured. All specimens have been deposited at the Centre for Behavioural Ecology and Evolution, College of Life Sciences, Hubei University, Wuhan, China (**CBEE**).

Abbreviations used. Morphological characters:

**ALE** anterior lateral eye;

**AME** anterior median eye;

**AME–ALE** distance between AME and ALE;

**AME–AME** distance between AME and AME;

**ALE–PLE** distance between ALE and PLE;

**AME–PME** distance between AME and PME;

**PLE** posterior lateral eye;

**PME** posterior median eye;

**PME–>PLE** distance between PME and PLE;

**PME–PME** distance between PME and PME;

## ﻿Taxonomy

**Family Agelenidae C.L. Koch, 1837**

**Subfamily Coelotinae F.O. Pickard-Cambridge, 1893**

**Genus *Yunguirius* B. Li, Zhao & S.Q. Li, 2023**

### ﻿ Yunguirius parvus

Taxon classificationAnimaliaAraneaeAgelenidae

Wei & Liu sp. nov.

D7452606-80E4-5FD0-BEAF-F344A3681201

https://zoobank.org/E2FDC5CF-C53D-47F2-9DA3-83F9C6AD20A1

Figs 2 , 3 , 10

#### Type material.

***Holotype*** ♀ (HBU-WM-24-001), 1♀ ***paratype*** (HBU-WM-24-002): China: Yunnan Province, Honghe Hani and Yi Autonomous, Gejiu County, Gejia Forest Park, 23.3893°N, 103.1254°E, elevation: 2045 m, 23.VIII.2020, M. Wei leg.

#### Etymology.

The specific epithet is taken from the Latin word *parvus*, meaning “small”, referring to the relatively small body type of new species; an adjective.

#### Diagnosis.

**Figure 1.**
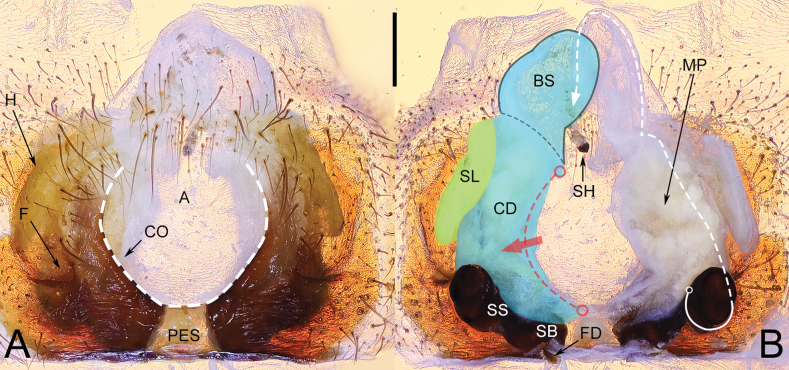
Epigyne and vulva of *Yunguiriusterebratus***A** epigyne, ventral view **B** vulva, dorsal view. Abbreviations: A = atrium; BS = blind sac; CD = copulatory duct; CO = copulatory opening; F = fold; FD = fertilization duct; H = hood; MP = mating plug; PES = posterior epigynal sclerite; SB = spermathecal base; SH = spermathecal head; SL = the secondary layer of copulatory duct; SS = spermathecal stalk. The white dashed line in A represents the margin of atrium and in B represents the spermathecal head. The black outline B shows the blind sac of the copulatory duct. The red dashed line and arrow in B indicate the opening of copulatory duct. The blue area indicates the copulatory duct, and the yellow area indicates the secondary layer of the copulatory duct. Scale bar: 0.50 mm.

**Figure 2.**
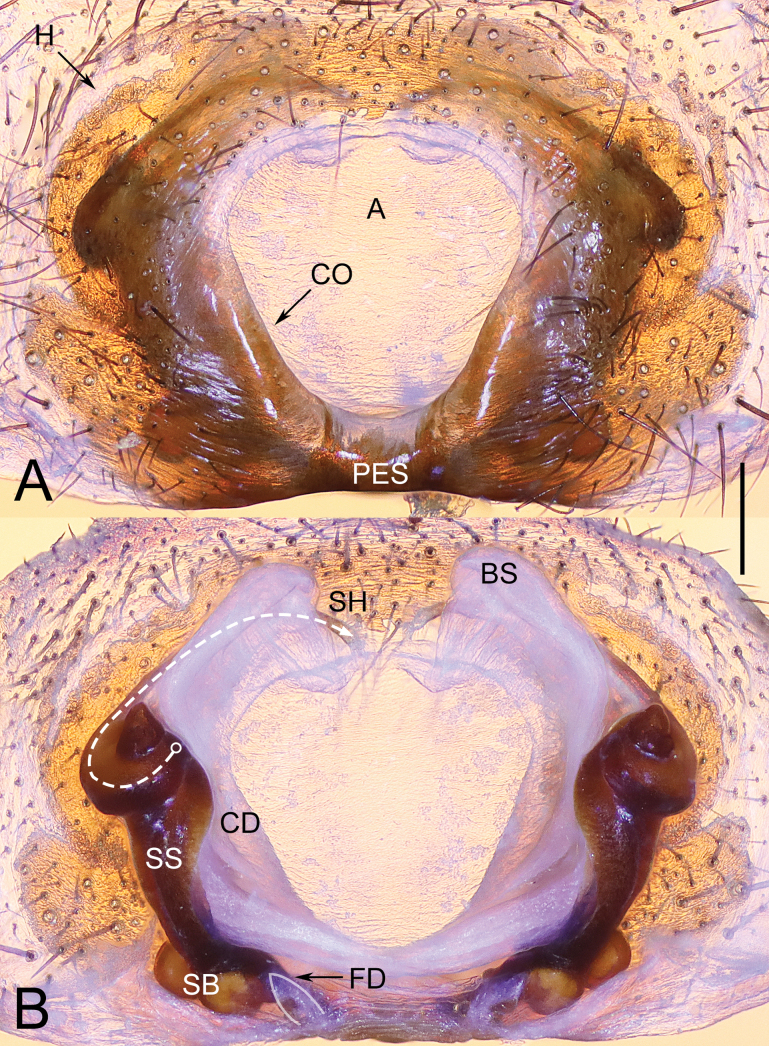
Epigyne of *Yunguiriusparvus* sp. nov. **A** epigyne, ventral view **B** vulva, dorsal view. Abbreviations: A = atrium; BS = blind sac; CD = copulatory duct; CO = copulatory opening; FD = fertilization duct; H = hood; PES = posterior epigynal sclerite; SB = spermathecal base; SH = spermathecal head; SS = spermathecal stalk. The white dashed line B indicates the spermathecal head, the white outline B indicates the fertilization duct. Scale bar: 0.50 mm.

**Figure 3.**
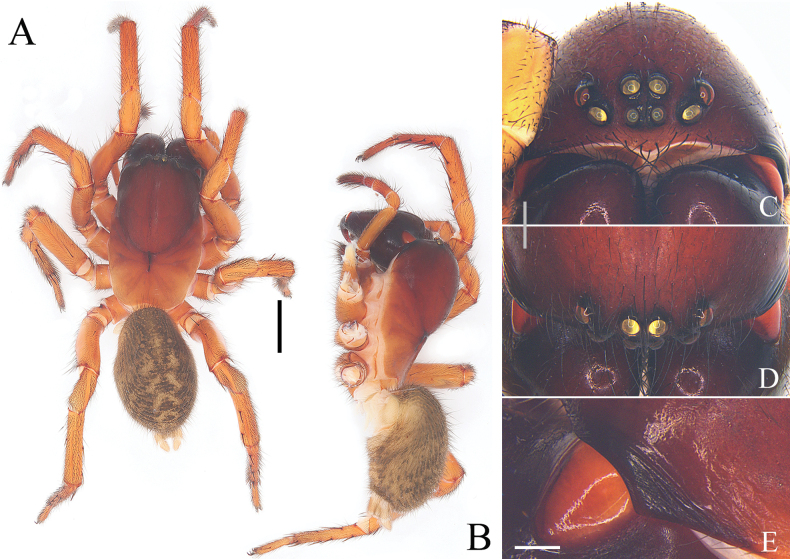
Characters of the female of *Yunguiriusparvus* sp. nov. **A** habitus, dorsal view **B** habitus, prolateral view **C** eye area, frontal view **D** eye area, dorsal view **E** cephalic rigion, lateral view. Scale bars: 2.00 mm (**A, B**); 0.50 mm (**C, D**); 0.25 mm (**E**).

**Figure 4.**
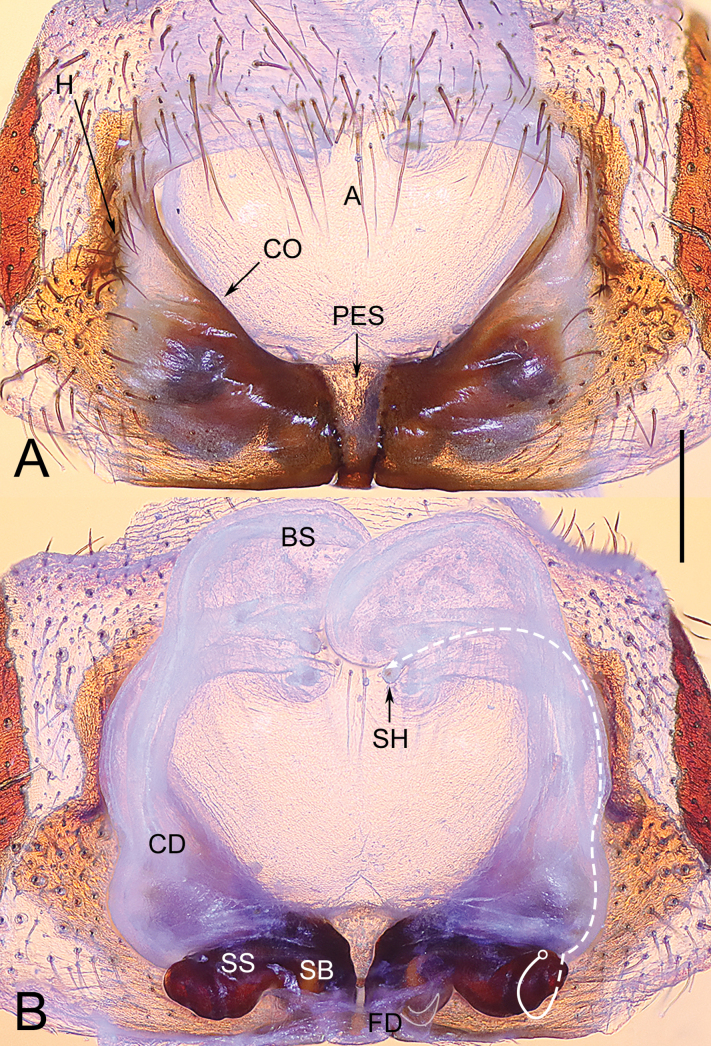
Epigyne of *Yunguiriustrigonus* sp. nov. **A** epigyne, ventral view **B** vulva, dorsal view. Scale bars: 1.00 mm. Abbreviations: A = atrium; BS = blind sac; CD = copulatory duct; CO = copulatory opening; FD = fertilization duct; H = hood; PES = posterior epigynal sclerite; SB = spermathecal base; SH = spermathecal head; SS = spermathecal stalk. The white dashed line B indicates the spermathecal head, the white outline B indicates the fertilization duct. Scale bar: 0.50 mm.

The females of *Yunguiriusparvus* sp. nov. resemble those of *Y.duoge* in 1) the atrium is subrounded with a complete anterior margin (Fig. 2A; fig. 2A in Li et al. 2023); 2) the openings of the copulatory ducts are wide, approximately half the circumference of the atrium (Fig. 2B; fig. 2B in Li et al. 2023); 3) the blind sacs of the copulatory ducts are extremely short, symmetrical, and separate (Fig. 2B; fig. 2B in Li et al. 2023). In other *Yunguirius* species, the atrium is non-subrounded (except in *Y.terebratus*) with an incomplete anterior margin (Figs 1A, 4A, 6A, 8A; fig. 245A in Zhu et al. 2017; figs 3A, 4A in Li et al. 2023), the copulatory openings are equal to or less than the length of the lateral margin of the atrium, and the blind sacs are asymmetrical and overlapping (Figs 1B, 4B, 6B, 8B; fig. 245B in Zhu et al. 2017; figs 3B, 4B in Li et al. 2023). However, *Y.parvus* sp. nov. can be differentiated from *Y.duoge* by 1) the absence of the fold (Fig. 2A), versus being present in the latter (Fig. 2A in Li et al. 2023); 2) the blind sac is shorter than the spermathecal stalk (Fig. 2B), versus being longer in the latter (Fig. 2B in Li et al. 2023); 3) the spermathecal stalk has a conch-shaped distal tip (Fig. 2B), versus being nearly round in the latter (Fig. 2B in Li et al. 2023).

#### Description.

**Female** (holotype) (Fig. 3). Carapace reddish brown. Cervical and radial groove distinct. Cephalic region moderately raised and wide, lateral margin with distinct furrows. Chelicerae with 3 promarginal teeth and 2 retromarginal teeth, condyle red. Sternum longer than wide. Abdomen pale yellow, with 5 chevron-shaped patterns, covered by hairs. Legs red. Total length 10.41. Carapace 5.85 long, 3.54 wide, cephalic region 3.12 wide. Abdomen 4.69 long, 3.10 wide. Eye size and interdistance: AME 0.19, ALE 0.23, PME 0.22, PLE 0.25; AME–AME 0.09, AME–ALE 0.14, AME–PME 0.09, ALE–PLE 0.05, PME–PME 0.06, PME–PLE 0.32. Leg measurements: Leg I 14.02 (1.80, 0.70, 3.53, 1.63, 2.78, 2.46, 1.39), leg II 12.40 (1.52, 0.69, 3.04, 1.48, 2.44, 2.23, 1.33), leg III 10.35 (1.38, 0.67, 2.43, 1.32, 1.68, 1.95, 1.08), leg IV 14.42 (1.66, 0.61, 3.50, 1.59, 2.91, 2.84, 1.41). Epigyne (Fig. 2). Epigynal teeth absent. Atrium centrally situated, subrounded, anterior margin complete. Epigynal sclerite small. Hoods weak, vertically oriented, situated laterally. Fold absent. Copulatory ducts openings broad, subequal to ½ the circumference of atrium, laterally originated, blind sacs short, symmetric, and untouched. Spermathecal bases consisted of 2 spherical chambers, spermathecal stalks long, with distal tips conch-shaped, spermathecal heads anteriorly originated, long and sclerotized. Fertilization ducts posteriorly situated.

**Male.** Unknown.

#### Distribution.

China (Yunnan).

### ﻿ Yunguirius trigonus

Taxon classificationAnimaliaAraneaeAgelenidae

Wei & Liu sp. nov.

177637C4-6F0F-513E-BBE2-2F864821751A

https://zoobank.org/8854F835-A7BA-448B-B3B2-B0921CB9E1A6

Figs 4 , 5 , 10

#### Type material.

***Holotype*** ♀ (HBU-WM-24-003): China: Chongqing City, Nanchuan District, Jinfo Mountain, 29.0489°N, 107.1279°E, elevation: 681 m, 30.IX.2021, T.X. Gu leg.

#### Etymology.

The specific epithet is derived from the Greek word “trigon”, meaning triangular and referring to the atrium and the posterior epigynal sclerite of the new species forming into a subtriangular pattern; an adjective.

#### Diagnosis.

The females of *Yunguiriustrigonus* sp. nov. resemble those of *Y.subterebratus* and *Y.wangqiqiae* sp. nov. in having a trapezoidal atrium, with the width longer than the length and the width at the widest point being three times longer than the narrowest point (Figs 4A, 6A; fig. 245A in Zhu et al. 2017), compared to being trapezoidal but with the width being shorter than the length, and the width of the widest point approximately being twice that of the narrowest point in *Y.ornatus* (Fig. 3A in Li et al. 2023), and being heart-shaped, pentagonal or subrounded in other *Yunguirius* species (Figs 1A, 2A, 8A; figs 2A, 4A in Li et al. 2023). However, *Y.trigonus* sp. nov. can be distinguished from the latter by the following characteristics: 1) the presence of a pair of long and linear hoods (Fig. 4A), versus having a pair of triangular hoods in the latter (Fig. 6A; fig. 245A in Zhu et al. 2017); 2) the short and slightly overlapping blind sacs of the copulatory ducts (Fig. 4B), versus being long and obviously overlapped in the latter (Fig. 6B; fig. 245B in Zhu et al. 2017); 3) the spermathecal stalks are relatively short and thick (Fig. 4B), versus being reduced in *Y.subterebratus* (fig. 245B in Zhu et al. 2017) or being subequal to half the width of the atrium in *Y.wangqiqiae* Wei & Liu, sp. nov. (Fig. 6B).

#### Description.

**Figure 5.**
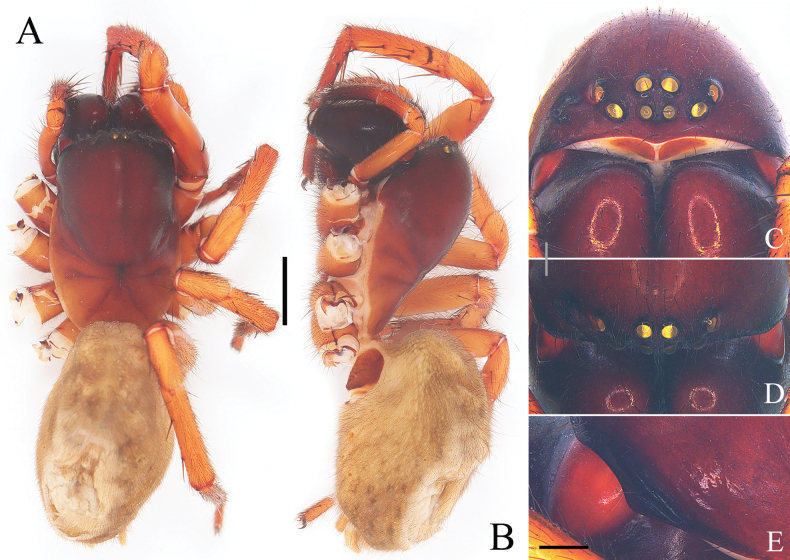
Characters of the female of *Yunguiriustrigonus* sp. nov. **A** habitus, dorsal view **B** habitus, prolateral view **C** eye area, frontal view **D** eye area, dorsal view **E** cephalic rigion, lateral view. Scale bars: 2.00 mm (**A, B**); 0.50 mm (**C, D**); 0.25 mm (**E**).

**Female** (holotype) (Fig. 5). Carapace reddish brown. Cervical and radial groove distinct. Cephalic region wide, moderately raised and wide, lateral margin with indistinct furrows. Chelicerae with 3 promarginal teeth and 2 retromarginal teeth, condyle red. Sternum longer than wide. Abdomen pale yellow, covered by hairs. Legs red. Total length 12.31. Carapace 5.83 long, 3.99 wide, cephalic region 3.49 wide. Abdomen 6.48 long, 3.68 wide. Eye size and interdistance: AME 0.18, ALE 0.27, PME 0.22, PLE 0.25; AME–AME 0.10, AME–ALE 0.17, AME–PME 0.16, ALE–PLE 0.10, PME–PME 0.11, PME–PLE 0.42. Leg measurements: Leg I 16.00 (2.12, 0.81, 3.97, 1.71, 3.39, 2.86, 1.57), leg II 14.71 (1.82, 0.82, 3.61, 1.66, 2.68, 2.74, 1.65), leg III 12.33 (1.55, 0.80, 2.99, 1.41, 1.94, 2.35, 1.45), leg IV 16.90 (1.87, 0.93, 4.13, 1.82, 3.41, 3.33, 1.61). Epigyne (Fig. 4). Epigynal teeth absent. Atrium centrally situated, trapezoidal, anterior margin incomplete, posterior margin short. Epigynal sclerite longer than wide. Hoods long, vertically oriented, situated laterally. Fold absent. Copulatory ducts broad, laterally originated, blind sacs short, distal tips slightly overlapped. Spermathecal bases normal, spermathecal stalks extended laterally, with distal tips conch-shaped, spermathecal heads reduced and membranous, distal tips visible. Fertilization ducts posteriorly situated.

**Male.** Unknown.

#### Distribution.

China (Chongqing).

### ﻿ Yunguirius wangqiqiae

Taxon classificationAnimaliaAraneaeAgelenidae

Wei & Liu sp. nov.

8CB5D448-FAB1-5233-A6B5-0DE67D1CFD86

https://zoobank.org/161CD48B-7C1E-4F46-BB59-0587C2B20AE0

Figs 6 , 7 , 10

#### Type material.

***Holotype*** ♀(HBU-WM-24-004), 1♀ ***paratype*** (HBU-WM-24-005): China: Yunnan Province, Zhaotong City, Weixin County, Houshan mountain, 27.8147°N, 104.8050°E, elevation: 1363 m, 1.X.2018, C.F. Tao and H.Y. Chen leg.

#### Etymology.

The specific name is dedicated to Ms Qiqi Wang, at the desire of Caifu Tao, who provided the holotype; a noun (name) in genitive case.

#### Diagnosis.

**Figure 6.**
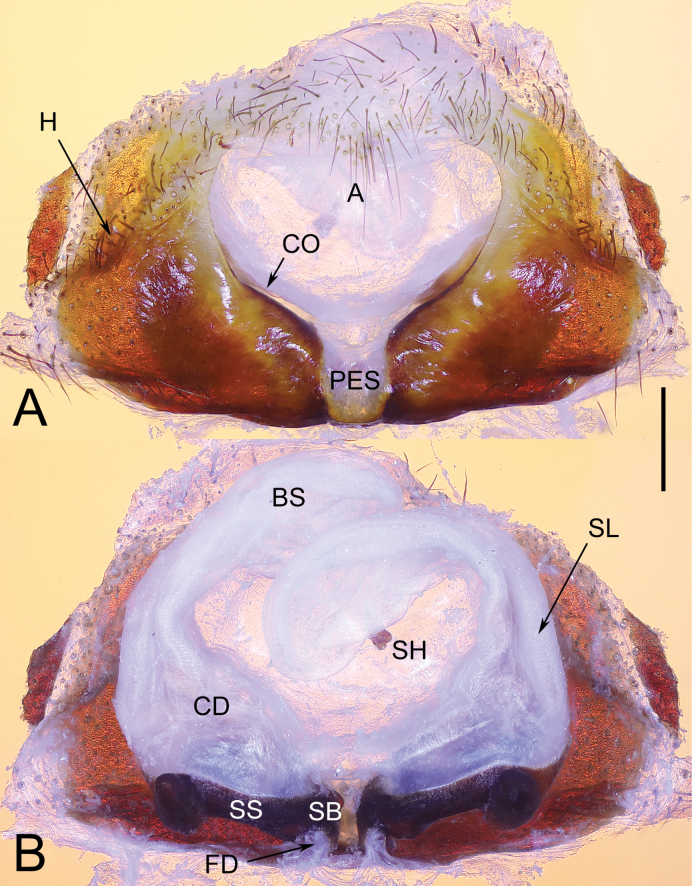
Epigyne of *Yunguiriuswangqiqiae* sp. nov. **A** epigyne, ventral view **B** vulva, dorsal view. Abbreviations: A = atrium; BS = blind sac; CD = copulatory duct; CO = copulatory opening; FD = fertilization duct; H = hood; PES = posterior epigynal sclerite; SB = spermathecal base; SH = spermathecal head; SL = the secondary layer of copulatory duct; SS = spermathecal stalk. Scale bar: 0.50 mm.

The females of *Yunguiriuswangqiqiae* sp. nov. resemble those of *Y.subterebratus* and *Y.terebratus* in that they have long blind sacs of the copulatory ducts, approximately equal to the length of the openings of the copulatory ducts, while the copulatory ducts are ventrally connected with the spermathecae (Figs 1B, 6B; fig. 245B in Zhu et al. 2017). In contrast, other species such as *Y.duoge*, *Y.parvus* sp. nov., *Y.trigonus* sp. nov. and *Y.xiangding* have short blind sacs, shorter than the length of the openings of the copulatory ducts (Fig. 2B, 4B; figs 2B, 4B in Li et al. 2023), or have long blind sacs but the copulatory ducts are dorsally connected with the spermathecae such as *Y.ornatus* and *Y.xiannushanensis* sp. nov. (Fig. 8; fig. 3B in Li et al. 2023). However, *Y.wangqiqiae* sp. nov. can be distinguished from the latter by the following characteristics: 1) the atrium is bowl-shaped, wider than long, and lacks the fold (Fig. 6A), versus being trapezoidal in *Y.subterebratus* (fig. 245A in Zhu et al. 2017) or being subrounded, with the width roughly equal to the length, and presenting the fold in *Y.terebratus* (Fig. 1A); 2) the copulatory ducts featuring only the prototype of the secondary layers (Fig. 6B), versus possessing advanced secondary layers in *Y.terebratus* (Fig. 1B); 3) the spermathecal stalks are long and extend laterally with conch-shaped distal ends (Fig. 6B), versus being extremely short in *Y.subterebratus* (fig. 245B in Zhu et al. 2017), and in *Y.terebratus*, they are long but extend obliquely upward, with large and round distal ends (Fig. 1B).

#### Description.

**Figure 7.**
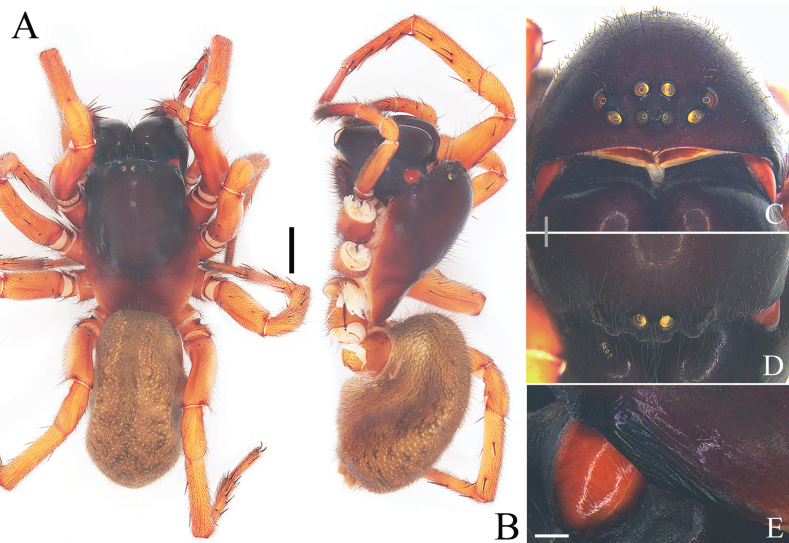
Characters of the female of *Yunguiriuswangqiqiae* sp. nov. **A** habitus, dorsal view **B** habitus, prolateral view **C** eye area, frontal view **D** eye area, dorsal view **E** cephalic region, lateral view. Scale bars: 2.00 mm (**A, B**); 0.50 mm (**C, D**); 0.25 mm (**E**).

**Female** (holotype) (Fig. 7). Carapace reddish brown. Cervical and radial groove distinct. Cephalic region moderately raised and wide, lateral margin with distinct furrows. Chelicerae with 3 promarginal teeth and 2 retromarginal teeth, condyle red. Sternum longer than wide. Abdomen pale yellow, with 5 chevron-shaped patterns, covered by hairs. Legs red. Total length 14.48. Carapace 7.51 long, 5.03 wide, cephalic region 4.28 wide. Abdomen 7.95 long, 4.80 wide. Eye size and interdistance: AME 0.22, ALE 0.31, PME 0.32, PLE 0.38; AME–AME 0.16, AME–ALE 0.20, AME–PME 0.19, ALE–PLE 0.11, PME–PME 0.09, PME–PLE 0.45. Leg measurements: Leg I 19.71 (2.53, 0.93, 4.98, 2.11, 4.08, 3.47, 1.96), leg II 17.82 (2.21, 0.86, 4.44, 1.93, 3.23, 3.28, 2.11), leg III 14.82 (2.00, 0.92, 3.67, 1.73, 2.44, 2.85, 1.61), leg IV 19.73 (2.31, 1.11, 4.87, 1.87, 3.84, 4.13, 1.84). Epigyne (Fig. 6). Epigynal teeth absent. Atrium large, bowl-shaped, anterior margin incomplete. Posterior epigynal sclerite weakly sclerotized and opalescent. Hoods weak, situated laterally. Fold absent. Copulatory ducts broad, laterally originated, slightly folded, with the prototype of the secondary layers; blind sacs long and with distal tips overlapped. Spermathecal base small; spermathecal stalk long, with distal tip conch-shaped and extended laterally; spermathecal head only remaining a sclerotized end. Fertilization ducts posteriorly situated.

**Male.** Unknown.

#### Distribution.

China (Guizhou, Yunnan).

### ﻿ Yunguirius xiannushanensis

Taxon classificationAnimaliaAraneaeAgelenidae

Wei & Liu sp. nov.

C5CAD8BE-B385-5107-87E5-554D0566E580

https://zoobank.org/40CEC348-B43B-4EB1-9491-103172F2690E

**Figure 8.**
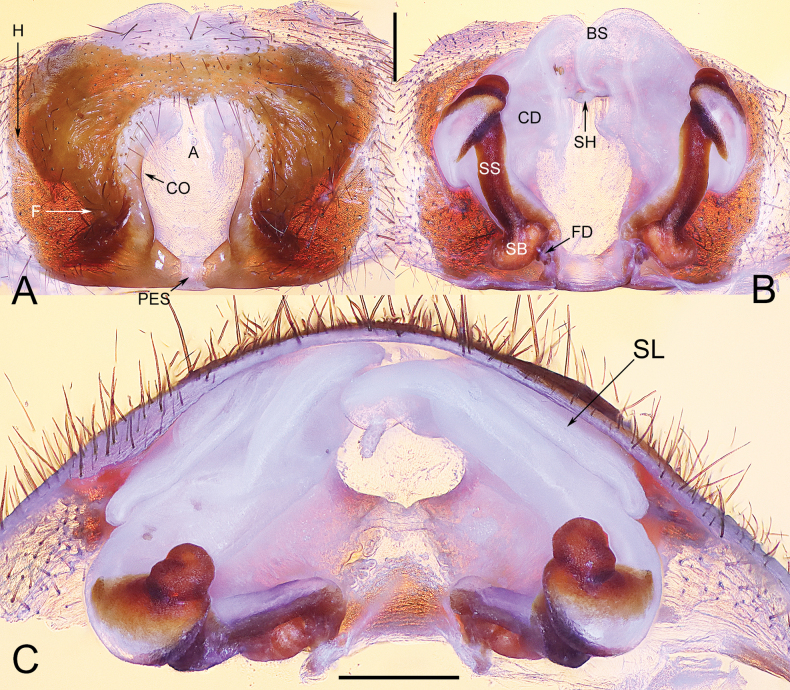
Epigyne of *Yunguiriusxiannushanensis* sp. nov. **A** epigyne, ventral view **B** vulva, dorsal view **C** vulva, apical view. Abbreviations: A = atrium; BS = blind sac; CD = copulatory duct; CO = copulatory opening; F = fold; FD = fertilization duct; H = hood; PES = posterior epigynal sclerite; SB = spermathecal base; SH = spermathecal head; SL = the secondary layer of copulatory duct; SS = spermathecal stalk. Scale bars: 0.50 mm.

**Figure 9.**
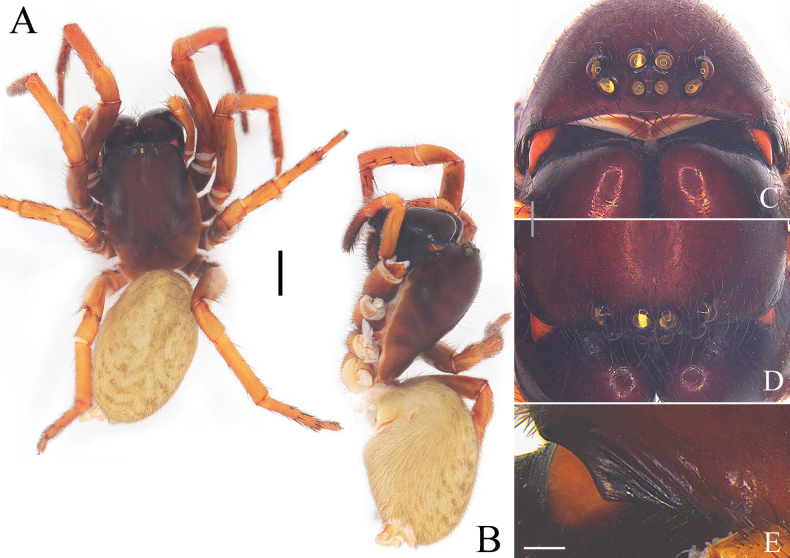
Characters of the female of *Yunguiriusxiannushanensis* sp. nov. **A** habitus, dorsal view **B** habitus, prolateral view **C** eye area, frontal view **D** eye area, dorsal view **E** cephalic region, lateral view. Scale bars: 2.00 mm (**A, B**); 0.50 mm (**C, D**); 0.25 mm (**E**).

**Figure 10.**
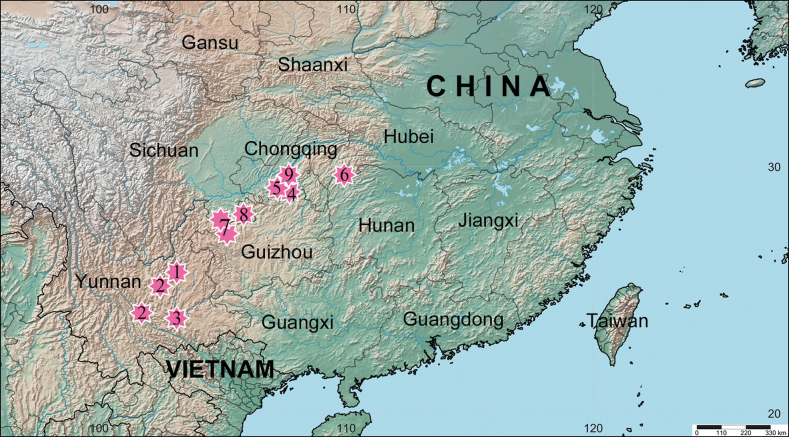
Distributions of the species of *Yunguirius*. 1 *Y.duoge* 2 *Y.ornatus* 3 *Y.parvus* sp. nov. 4 *Y.subterebratus* 5 *Y.terebratus* 6 *Y.trigonus* sp. nov. 7 *Y.wangqiqiae* sp. nov. 8 *Y.xiangding* 9 *Y.xiannushanensis* sp. nov.

**Figure 11.**
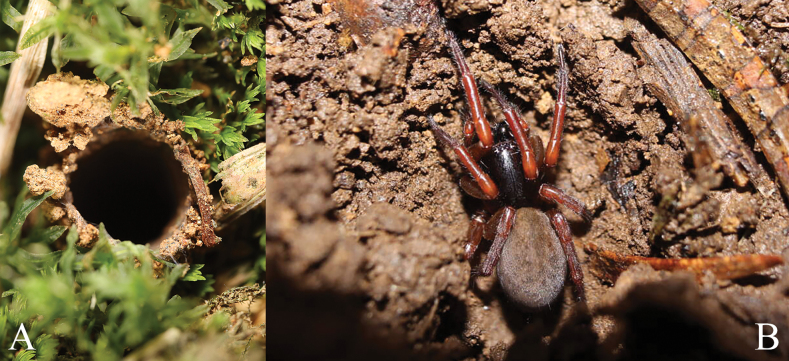
Photos of the nest and the living female of *Yunguiriusxiannushanensis* sp. nov. **A** opening of the tube nest **B** living female.

Figs 8 , 9 , 10 , 11

#### Type material.

***Holotype*** ♀ (HBU-WM-24-006): China: Chongqing City, Wulong District, Xiannu Mountain, 29.4508°N, 107.7280°E, elevation: 1951 m, 15.IX.2021, T.X. Gu leg.

#### Etymology.

The new species is named after the type locality, Xiannu Mountain; an adjective.

#### Diagnosis.

The females of *Yunguiriusxiannushanensis* sp. nov. resemble those of *Y.ornatus* in 1) the atrium is relatively small, less than 1/3 the width of the epigyne, with a reduced anterior margin (Fig. 8A; fig. 3A in Li et al. 2023); 2) the connection of the copulatory duct and the spermatheca presents dorsally (Fig. 8B; fig. 3B in Li et al. 2023). While in other *Yunguirius* species, the atrium exceeding 1/3 the width of the epigyne, with the anterior margin complete (*Y.duoge* and *Y.parvus* sp. nov., fig. 2A; fig. 2A in Li et al. 2023) or incomplete (*Y.subterebratus*, *Y.terebratus*, *Y.trigonus* sp. nov., *Y.wangqiqiae* sp. nov. and *Y.xiangding*, figs 1A, 4A, 6A; fig. 245A in Zhu et al. 2017; fig. 4A in Li et al. 2023), and the connection of the copulatory duct and the spermatheca presents ventrally (Figs 1B, 2B, 4B, 6B; fig. 245B in Zhu et al. 2017; figs 2B, 4B in Li et al. 2023). *Y.xiannushanensis* sp. nov. can be distinguished from *Y.ornatus* by the following characteristics: 1) the atrium is pentagonal (Fig. 8A), versus being trapezoidal in *Y.ornatus* (Fig. 3A in Li et al. 2023); 2) the posterior epigynal sclerite is reduced and thin, roughly a quarter of the width of the atrium (Fig. 8A), versus being more substantial and about equal to the width of atrium in *Y.ornatus* (Fig. 3A in Li et al. 2023); 3) the copulatory ducts are folded, and with distinct secondary layer (Fig. 8B), versus being monolayered in *Y.ornatus* (Fig. 3B in Li et al. 2023); 4) the spermathecal bases are large, twice as wide as the stalks, the spermathecal stalks have conch-shaped distal tips, and the spermathecal heads are membranous and only the distal tips are visible (Fig. 8B); in contrast, in *Y.ornatus*, the spermathecal bases are relatively small, slightly wider than the stalks, the distal tips of the stalks are normal, and the spermathecal heads are long and sclerotized (Fig. 3B in Li et al. 2023).

#### Description.

**Female** (holotype) (Fig. 9). Carapace reddish brown. Cervical and radial groove distinct. Cephalic region moderately raised and wide, lateral margin with distinct furrows. Chelicerae with 3 promarginal teeth and 2 retromarginal teeth, condyle red. Sternum longer than wide. Abdomen pale yellow, with 5 chevron-shaped patterns, covered by hairs. Legs red. Total length 13.20. Carapace 6.40 long, 4.25 wide, cephalic region 3.70 wide. Abdomen 7.14 long, 4.36 wide. Eye size and interdistance: AME 0.19, ALE 0.25, PME 0.25, PLE 0.28; AME–AME 0.12, AME–ALE 0.18, AME–PME 0.10, ALE–PLE 0.05, PME–PME 0.13, PME–PLE 0.34. Leg measurements: Leg I 17.18 (2.26, 0.75, 4.27, 1.91, 3.57, 3.20, 1.69), leg II 15.25 (1.94, 0.74, 3.79, 1.70, 3.02, 2.79, 1.67), leg III 12.68 (1.64, 0.79, 3.15, 1.45, 2.10, 2.39, 1.40), leg IV 17.77 (1.94, 0.95, 4.40, 1.88, 3.57, 3.49, 1.70). Epigyne (Fig. 8). Epigynal teeth absent. Atrium relatively small, pentagonal, anterior margin reduced. Epigynal sclerite small, opalescent. Hoods weak, vertically oriented, situated laterally. Fold distinct, triangular. Copulatory ducts broad, laterally originated, folded into 2 layers, and connected with spermathecae ventrally; blind sacs broad and short. Spermathecal base bean-shaped and twice wider than width of spermathecal stalk; spermathecal stalk long, with distal tip conch-shaped; spermathecal head reduced, only remaining a membranous tip on the distal tip of blind sac. Fertilization ducts posteriorly situated.

**Male.** Unknown.

#### Distribution.

China (Chongqing).

#### Notes.

Our fieldwork indicates that these new *Yunguirius* species inhabit tube nests with round openings dug into soil, moss, or rotten wood of high humidity, rather than constructing funnel webs beneath rocks or crevices like some other common agelenid spiders. A further study may be required to determine the origins of the burrowing behavior of these spiders.

## Supplementary Material

XML Treatment for Yunguirius parvus

XML Treatment for Yunguirius trigonus

XML Treatment for Yunguirius wangqiqiae

XML Treatment for Yunguirius xiannushanensis

## ﻿Additional information

### Author ORCIDs

Mian Wei https://orcid.org/0000-0001-7348-8885

Kai Wang https://orcid.org/0000-0002-9877-5140

### Data availability

All of the data that support the findings of this study are available in the main text.
